# Molecular Mechanisms of Glucocorticoid Action in Migraine: A Systematic Review of Neuroinflammatory and Vascular Pathways

**DOI:** 10.7759/cureus.96074

**Published:** 2025-11-04

**Authors:** Luciano da Silva Lopes, Abouch Krymchantowski, Carla Jevoux, Ana Gabriela Krymchantowski, Camila M Maria Coelho de Moura, Adriana A Soares, Marco Antônio Pereira de Sá, Sabrina Nayara de Araújo Val, Renato Mendes dos Santos, Raimundo Pereira Silva-Néto

**Affiliations:** 1 Department of Biophysics and Physiology, Universidade Federal do Piauí, Teresina, BRA; 2 Department of Neurology, Headache Center of Rio, Rio de Janeiro, BRA; 3 Department of Neurology, Universidade Federal do Piauí, Teresina, BRA; 4 Department of Neurology, Universidade Federal do Delta do Parnaíba, Parnaíba, BRA; 5 Department of Sports, Secretaria de Educação e Desporto de Roraima, Amajari, BRA; 6 Department of Medicine, Universidade Federal do Piauí, Teresina, BRA

**Keywords:** calcitonin gene-related peptide, glucocorticoids, matrix metalloproteinase-9, migraine, neuroinflammation

## Abstract

Migraine is a prevalent neurological disorder with complex pathophysiology that involves neurogenic inflammation, vasodilation, and hypersensitization of the trigeminovascular system. Recently, glucocorticoids have attracted attention as adjunctive treatments for migraine, mainly in refractory or recurrent attacks. Although they are traditionally recognized for their anti-inflammatory effects, the specific molecular mechanisms by which glucocorticoids alleviate migraine symptoms are not yet fully understood. In this study, we systematically reviewed the literature from 2004 to 2024 to evaluate the molecular mechanisms by which glucocorticoids exert their therapeutic effects in migraine. The analysis focused mainly on their impact on neuroinflammatory mediators, the integrity of the blood-brain barrier, and nociceptive signaling. Results from 26 selected articles showed that glucocorticoids decreased the expression of matrix metalloproteinase-9 (MMP-9), inhibited nitric oxide synthesis, and reduced calcitonin gene-related peptide (CGRP) levels, all of which are key agents implicated in migraine pathogenesis. Furthermore, glucocorticoids modulate transcription factors such as nuclear factor kappa-light-chain-enhancer of activated B cells (NF-κB) and activator protein 1 (AP-1), reduce the production of pro-inflammatory cytokines like interleukin-1 beta (IL-1β) and tumor necrosis factor-alpha (TNF-α), and limit prostaglandin synthesis via cyclooxygenase-2 (COX-2) inhibition. These pathways suppress central and peripheral sensitization, which provides symptom relief. Our findings suggest that glucocorticoids play a more significant role in migraine management than previously appreciated. This influences neuroimmune responses and vascular permeability. These insights state the therapeutic potential of glucocorticoids beyond general inflammation control and encourage more targeted clinical use. This is especially true in cases with neuroinflammatory features.

## Introduction and background

Migraine is a common and disabling neurological disorder that affects millions of people worldwide. Along with tension headaches, it is one of the most common primary headache disorders, posing a significant public health challenge due to its widespread prevalence and significant impact on quality of life, productivity, and the economy [1]. It can have an impact on relationships, daily tasks, and work life. It is also a leading cause of years lived with disability. Furthermore, approximately 2% to 3% of the population has chronic migraine, the most severe form of the disease, with a higher incidence among young women and adolescents [2]. There is evidence of several triggers for headache attacks, such as stress, hormonal changes, sleep deprivation, physical exertion, sensory stimuli, prolonged fasting, and the consumption of certain foods [3-8].

In recent decades, several new classes of drugs have been proposed for the acute and preventive treatment of migraine patients [9-12]. Since the synthesis of sumatriptan, triptans combined with nonsteroidal anti-inflammatory drugs have been the main option for controlling headache attacks [13]. For prevention, several chemical groups of drugs have been used, including beta-blockers, calcium channel blockers, tricyclic antidepressants, and neuromodulators. This variety of drugs, with different mechanisms of action, reflects a partial but growing understanding of the pathophysiological mechanisms of the disease, resulting in varying levels of efficacy and tolerability for these drugs [9-12].

The recent approval of anti-CGRP therapies represents a significant advance in migraine treatment due to the modulation of signaling pathways crucial to pain triggering, with evidence of the role of the trigeminal-vascular system and calcitonin gene-related peptide (CGRP) in this process. Initially, monoclonal antibodies (erenumab, galcanezumab, fremanezumab, and eptinezumab) that bind to CGRP or its receptor and are used in the preventive treatment of migraine emerged; subsequently, gepants (rimegepant, atogepant, zavegepant, and ubrogepant), small-molecule antagonists of the CGRP receptor, are used in headache attacks or for migraine prevention. In addition to anti-CGRP therapies, there is lasmiditan, a selective 5-hydroxytryptamine 1F (5-HT1F) receptor, that also participates in the pathophysiology of migraine by decreasing vasodilation [9-12].

Glucocorticoids can be used to treat migraine attacks, particularly in severe cases resistant to other treatments, cases associated with overuse of analgesics, or frequent and disabling migraines. They have anti-inflammatory properties and can help reduce the duration, intensity, and recurrence of new headache attacks when administered intravenously and in combination with other medications [14,15].

Consequently, this study seeks to analyze the current literature and investigate the precise molecular pathways by which glucocorticoids exert their therapeutic effects in migraine. By better understanding these mechanisms, we hope to support more targeted and effective use of glucocorticoids in the clinical treatment of migraine.

## Review

Methodology

This study followed the Preferred Reporting Items for Systematic Reviews and Meta-Analyses (PRISMA) guidelines to review the molecular mechanisms of action of glucocorticoids in migraine published between 2004 and 2024. The search strategy was carried out in online databases such as Latin American and Caribbean Health Sciences Literature (LiLacs), Scientific Electronic Library Online (SciELO), Biblioteca Regional de Medicina (Latin American and Caribbean Center on Health Sciences Information) (Bireme), ScienceDirect, and Public/Publisher MEDLINE (standard biomedical database) (PubMed), using the descriptors "migraine," "migraine medications," "glucocorticoids and migraine," and "acute treatment of migraine." Articles in English that addressed the discussion of glucocorticoids and migraine were included. The PubMed search was conducted on January 15, 2024, using the terms (‘migraine’ [MeSH Terms] OR ‘migraine disorders’) AND (‘glucocorticoids’ OR ‘corticosteroids’ OR ‘dexamethasone’ OR ‘prednisolone’). Similar strategies were adapted for SciELO, LILACS, BIREME, and ScienceDirect. The complete search strings for each database are provided in Table 3 in Appendices, in compliance with PRISMA requirements. 

Table 1 provides the characteristics of the included studies.

**Table 1:** Distribution of the 26 articles used in this review ED: emergency departments; NSAIDs: non-steroidal anti-inflammatory drugs; NA: not applicable; CGRP: calcitonin gene-related peptide; PACAP: pituitary adenylate cyclase-activating polypeptide; CSD: cortical spreading depression; MMP-9: matrix metalloproteinase-9; TIMP-1: tissue inhibitor of metalloproteinases-1; BBB: blood-brain barrier; IL-1β: interleukin-1β.

Category	Authors & Year	Study Type	Sample	Key Findings
Clinical studies (RCTs, observational)	Krymchantowski et al., 2020 [16]	Retrospective, cross-sectional, observational	n=84	Most frequently used medications in ED for migraine were dipyrone (89.3%), NSAIDs (57.1%), opioids (51.1%), metoclopramide (29.8%), dexamethasone (28.6%), chlorpromazine (13.1%), and subcutaneous sumatriptan (7.1%).
	Silva-Néto et al., 2014 [17]	Prospective, comparative	n=400	Osmophobia occurred in 86% of migraine patients versus 6% in tension-type headache patients.
	Karademir et al., 2022 [18]	Observational, case–control	n=75	TIMP-1 levels were lower in migraine patients; the MMP-9/TIMP-1 ratio was significantly higher.
	Tfelt-Hansen et al., 2009 [19]	RCT, double-masked, placebo-controlled	n=15	Prednisolone reduced the frequency and severity of nitric oxide–induced migraine symptoms.
	Neeb et al., 2014 [20]	Clinical observational	n=15	Corticosteroid treatment reduced CGRP levels and increased melatonin secretion in cluster headache patients.
Meta-analyses & systematic reviews	Colman et al., 2008 [14]	Meta-analysis	n=738	Single parenteral dexamethasone with standard abortive therapy reduced headache recurrence by 26% within 24–72 h.
	Huang et al., 2013 [21]	Meta-analysis	n=905	Parenteral dexamethasone showed greater efficacy in reducing recurrent moderate/severe headaches.
Basic science/experimental studies	Gursoy-Ozdemir et al., 2004 [22]	In vivo experimental	NA	Induction of CSD increased MMP-9 activity, disrupted BBB proteins, and raised vascular permeability.
	Kim, 2008 [23]	In vivo experimental	NA	Methylprednisolone downregulated MMP-9, reversed by RU486; COX-2 and TNF-α were unaffected.
	Neeb et al., 2016 [24]	In vitro experimental	NA	Methylprednisolone reduced IL-1β–stimulated CGRP release, suggesting an anti-inflammatory mechanism.
	Cuesta et al., 2002 [25]	In vitro experimental	NA	Substance P and CGRP increased secretion of IL-1β, IL-6, and TNF-α, supporting a role in neurogenic inflammation.
Narrative reviews	Silvestro et al., 2023 [11]	Narrative review	NA	Comprehensive review of emerging pharmacological targets for migraine treatment.
	Giuliano et al., 2012 [15]	Narrative review	NA	Summarized clinical trial and meta-analysis data supporting adjuvant dexamethasone in migraine therapy.
	Villar-Martinez et al., 2022 [26]	Narrative review	NA	Emphasized individualized treatment and thorough clinical anamnesis in headache care.
	Russo et al., 2023 [27]	Narrative review	NA	Reviewed the regulation and pharmacology of CGRP and its receptor.
	Ferrari, 2022 [28]	Narrative review	NA	Outlined migraine therapy: NSAIDs/analgesics for mild attacks, triptans/5HT1B/1D agonists for moderate/severe.
	Edvinsson, 2021 [29]	Narrative review	NA	Discussed CGRP’s role in migraine pathophysiology and targeted therapies.
	Kamm, 2022 [30]	Narrative review	NA	Investigated CGRP levels in jugular/peripheral blood and migraine contribution.
	Chan et al., 2019 [31]	Narrative review	NA	Reviewed CGRP and PACAP roles via clinical, imaging, and preclinical studies.
	Jiménez-Jiménez et al., 2024 [32]	Narrative review	NA	Proposed oxidative stress–inflammation interactions in migraine pathogenesis.
	Gupta, 2009 [33]	Narrative review	NA	Suggested cerebral blood flow changes and CAD–MMP-9 association remain hypothetical.
	Rhen and Cidlowski, 2005 [34]	Narrative review	NA	Explained genomic and non-genomic anti-inflammatory actions of glucocorticoids.
	Vandewalle et al., 2018 [35]	Narrative review	NA	Detailed therapeutic mechanisms of glucocorticoids: gene regulation, immune modulation, signaling.
	Williams, 2018 [36]	Narrative review	NA	Reviewed pharmacokinetics, pharmacodynamics, and therapeutic applications of corticosteroids.
	Kursun, 2021 [37]	Narrative review	NA	Focused on neuroinflammation and the inflammasome complex in migraine.
	Karsan, 2023 [38]	Narrative review	NA	Discussed molecular migraine mechanisms, especially nitric oxide synthase and neuropeptides.

Inclusion criteria consisted of original research articles and high-quality narrative or systematic reviews addressing the use of glucocorticoids or molecular mechanisms in migraine management. Exclusion criteria included editorials, commentaries, letters, case reports, duplicated records, and articles lacking accurate or relevant scientific data.

**Figure 1:**
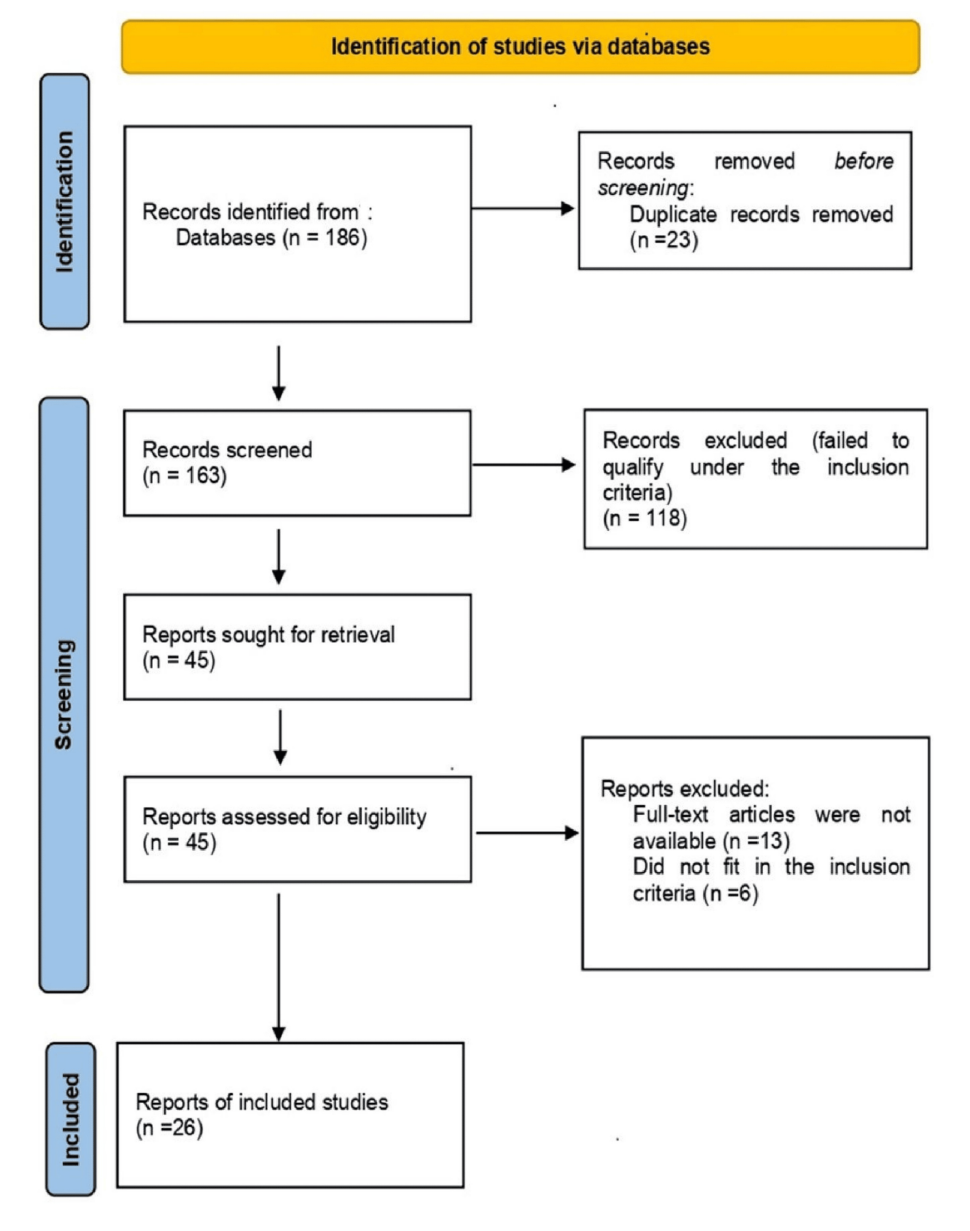
Flowchart of the selection of articles included in the review

Two independent reviewers conducted the selection, with disagreements resolved by consensus or consulting a third reviewer. Results were qualitatively synthesized, and, where possible, data were combined to identify patterns and differences in reported results. As the paper is a literature review, submitting the study to the research ethics committee was unnecessary, but all sources consulted were cited in the work. Since this study involved the analysis of publicly available data and did not include human subjects, institutional ethics approval was not required. All sources are duly cited. Figure 1 depicts the flowchart of the search sequence leading up to the inclusion of articles.

Evaluation of the quality of the studies was carried out according to the format proposed by Murad et al. [39] which includes four domains: selection, verification, causality, and communication (Table 2). The methodological quality was rated in general based on the recommendations, and all the recognized studies in supplementary Table 4 in the Appendices were satisfactory in terms of methodological quality.

**Table 2:** Evaluation of the methodological quality of the articles included in this review

Domain	Explanatory questions	Methodological quality
Selection	Does the patient(s) represent(s) the whole experience of the investigator (center) or is the selection method unclear to the extent that other patients with similar presentation may not have been reported?	Yes
Ascertainment	Was the exposure adequately ascertained?	Yes
	Was the outcome adequately ascertained?	Yes
Causality	Were there other alternative causes that may explain why the observation was ruled out?	Yes
	Was there a challenge/rechallenge phenomenon?	Yes
	Was there a dose–response effect?	No
	Was follow-up long enough for outcomes to occur?	No
Reporting	Is the case(s) described with sufficient detail to allow other investigators to replicate the research or to allow practitioners to make inferences related to their own practice?	Yes

The methodological quality of included studies was assessed using validated tools according to study design. The single randomized controlled trial (RCT) was evaluated with the Cochrane Risk of Bias 2 (RoB 2) tool, covering five domains. Risk Of Bias In Non-randomized Studies of Interventions (ROBINS-I) was applied to non-randomized observational studies, addressing seven bias domains. The Systematic Review Centre for Laboratory Animal Experimentation’s (SYRCLE) Risk of Bias tool was used for animal studies, while in vitro experiments were narratively appraised. Scale for the Assessment of Narrative Review Articles (SANRA) was employed to assess narrative reviews across six domains. Systematic reviews (SRs) and meta-analyses (MAs) were examined using A Measurement Tool to Assess Systematic Reviews 2 (AMSTAR-2), encompassing 16 domains. Results are presented in Supplementary Table 5 (RoB 2, ROBINS-I, SYRCLE, SANRA) and Table 6 (AMSTAR-2).

Results

The initial search of the systematic database retrieved a total of 186 articles. After removing duplicate results (n = 26), a total of 163 articles were left. The titles and abstracts were then filtered on relevance grounds, and 118 articles were excluded because they failed to qualify under the inclusion criteria. Out of the total of 45 articles, 13 full-text articles were not available, and the total number of available full-text articles is 32 to be critically assessed. After full-text evaluation, six more articles were removed as they did not fit the inclusion criteria. Twenty-six articles were used in this qualitative synthesis and analysis. The article selection process is visually summarized in the flowchart presented in Figure 1. Results are presented in Supplementary Tables 3-6 (RoB 2, ROBINS-I, SYRCLE, SANRA, and AMSTAR-2).

Pathophysiology of Migraine and the Therapeutic Role of Glucocorticoids

Migraine treatment landscape and glucocorticoid applications: The management of migraine involves different therapeutic approaches, utilizing various pharmacological categories for both preventative treatment and the management of acute migraine attacks in this primary neurological disorder. Intelligent choice of medications is the key to effective management of mood increases, the progression of chronic pain, and the decline of a patient's quality of life [16]. 

Glucocorticoids such as dexamethasone have become candidates that may have the ability to limit the recurrences of migraine in emergency departments, whereas they are not considered primary abortive agents. Clinical evidence has revealed that symptom recurrence in up to 49 percent of treated patients occurs within a 72-hour period. Several things affect the effectiveness of treatment, such as the abortive medication used, their age, sex, and severity of headache at initial stages. Most predictors are attributed to age above 35 years, female gender, and the degree of severity of the headache. But with migraine, there are inconsistent findings regarding glucocorticoids, where the efficacy is 30 percent or less compared to placebo [17,22]. 

Similarly, Woldeamanuel et al. conducted a 65-year systematic review and revealed that single-dose parenteral dexamethasone, commonly 10 mg intravenous (IV), contributed to a median absolute risk reduction of 11% in 72-hour recurrence and demonstrated particular benefit when used adjunctively in patients with higher migraine-related disability or status migrainosus [40]. Furthermore, Woldeamanuel et al. discovered markedly increased serum and cerebrospinal fluid (CSF) cortisol and corticosterone concentrations in chronic migraine patients relative to episodic migraineurs and healthy controls, indicating that endogenous glucocorticoids may function as biomarkers for migraine chronification and assist in evaluating treatment responsiveness [41].

Molecular Mechanisms Linking Migraine Pathophysiology to Glucocorticoid Action

Trigeminovascular system activation: Migraine is a severe, complex, multisymptomatic disorder with various non-pain symptoms. Their pathophysiologic mechanism is the induction of the trigeminovascular system, which contains trigeminal nerve fibers that provide the cranial and meningeal vessels. This process mediates the release of neuropeptides, including calcitonin gene-related peptides (CGRPs), substance P, and neurokinin A, which enhance vasodilation and contribute to neurogenic inflammation involved in pain development. The disease and patients' quality of life are also associated with symptoms such as nausea, vomiting, photophobia, phonophobia, osmophobia, allodynia, and vertigo [18,27]. 

Nausea and vomiting are among the most common symptoms of migraine, negatively affecting quality of life, and their occurrence is linked to the activation of brain areas associated with dopaminergic and serotonin transmission. Osmophobia, which is intolerance to smell and especially to perfumes, is related to the central sensitization of olfactory pathways [28]. It is a common phonophobia condition, and it could be associated with a probability of hearing loss and sound sensitivity. Increased pain sensitivity in the cranial and extracranial areas, which is denoted by allodynia, is associated with trigeminal neuron sensitization. Vertigo often accompanies migraine, one of the main causes of patients' incapacitation, and vestibular disorders are associated with it [18,27]. Similarly, Al-Hassany et al. highlighted that activation of the trigeminovascular system, particularly the release of CGRP, substance P, and neurokinin A, underpins migraine pathophysiology and contributes to peripheral and central sensitization processes that exacerbate migraine symptoms and reduce quality of life [42].

The Central Role of CGRP in Migraine Pathophysiology

Studies show that plasma and other fluids contain remarkable levels of CGRP in migraine attacks, a fact that shows its role in the processes of both vasodilation and sensitization of nociceptors, factors that activate pain production. Furthermore, the trigeminal system releases CGRP, which triggers inflammatory processes that sustain pathways for pain development. Intravenous infusion of CGRP in predisposed patients provokes migraine attacks, directly implicating it in the trigeminovascular sensitization process [27-30]. 

CGRP works in a specific receptor, mainly the CGRP-R1, which is located mostly in the neurons of the trigeminus and the cells of the vascular smooth muscle. Activation of the receptors causes vasodilation and the modulation of neural transportation of pain. Based on this information, treatment using monoclonal antibodies against CGRP or its receptors has been shown to effectively prevent migraines and reduce the frequency of headache attacks. The given antibodies also block the role of CGRP without affecting other essential physiological processes, which proves the significant role of CGRP in the pathogenesis of migraines [27-30]. Similarly, Thomas et al. demonstrated that CGRP signaling disrupts meningeal lymphatic vessel function by inducing rearrangement of gap junction proteins, thereby reducing CSF drainage and promoting neuroinflammation in migraine models [43].

Migraine occurs very frequently in women, particularly after certain ages, indicating that hormones play a significant role. The estrogens, hormones that vary throughout menstrual periods, relate to the CGRP regulation, increasing its secretion. Research indicates that women possess greater basal CGRP levels and responses to CGRP-releasing agents, which may be the reason why women are more afflicted by the disease [27].

Numerous anatomical locations and physiological systems of migraine are points of action for CGRP. The trigeminovascular system is directed peripherally, in which the meningeal artery vasodilates, producing a rise in tissue pressure and subsequent stimulation of pain-sensitive receptors. CGRP also leads to the release of other inflammatory mediators, which worsen peripheral nociceptor sensitization. CGRP is released by terminals of the brainstem, especially the trigeminal nucleus caudal area of the central nervous system, which proves to be the most important site where the nerve can modulate and amplify brain pain [30,31].

Across the second- and first-order neurons of this nucleus, this effect is achieved by increased production of pain signals, which lead to typical cramping pain and the sensation of pain hypersensitivity to sensory stimuli (photophobia, phonophobia, and osmophobia). The interaction between peripheral and central CGRP actions forms a bidirectional loop in which peripheral CGRP stimulates the trigeminovascular system, sending signals to the brainstem that propagate central sensitization. Such a repeated process enhances responsiveness to painful stimuli, leading to chronicity in a few patients [30,31]. There is a bidirectional interaction between peripheral and central CGRP actions. Peripheral CGRP activates the trigeminovascular system, sending signals to the brainstem that propagate central sensitization. This feedback loop heightens pain responsiveness and may lead to chronic migraine in some patients [44].

Additional Pathophysiological Mechanisms

Pituitary adenylate cyclase-activating polypeptide (PACAP): The significant peptide in the pathophysiology of migraine is PACAP. Preclinical reports have shown PACAP to play a critical role in the transmission of pain signals, with reports pinpointing its role in modulating C fiber responses implicated in nociceptive pain transmission. Moreover, released PACAP could enhance the release of CGRP within the trigeminocervical system, and PACAP might exert this effect by acting on the PAC1 receptor. Specific monoclonal antibodies enhance the enforcement of this receptor by inhibiting nociceptive activity [32]. Similarly, Guo et al. emphasized that PACAP38 plays a distinct and significant role in migraine pathophysiology, operating independently from the CGRP pathway and offering a potential therapeutic target, particularly for patients unresponsive to CGRP-based treatments. Nonetheless, mechanisms that encourage the release of CGRP are not fully clarified [32,45].

Oxidative stress: In the pathophysiology of migraine, free radicals produced by oxidative stress play a significant role in the production of thiobarbituric acid-reactive substances, malonyldialdehyde, and nitric oxide (NO). Research shows that a low total antioxidant capacity exists in migraine patients, and the activities of superoxide dismutase are usually low during a headache attack. Oxidative stress can facilitate inflammatory pathway activation and help in the development of neuronal dysfunction that may result in nervous system sensitization [32]. Similarly, Gupta suggested that oxidative stress may not only trigger migraine but also interact synergistically with inflammation to sustain attacks [33]. The interaction occurring is complex in its nature, and it can lead to headache attacks and other related migraine symptoms [32]. 

Matrix metalloproteinases: Matrix metalloproteinase-9 (MMP-9) is a proteolytic protein that degrades extracellular matrix components, such as collagen and fibronectin. This degradation compromises blood-brain barrier (BBB) ​​permeability. The enzymes in migraine are correlated with high transient BBB permeability and neurogenic inflammation, two mechanisms that are closely implicated in migraine pathology. Research shows that plasma MMP-9 level rises considerably in cases of migraine attacks, mostly during headache stages and after termination. Such augmentation of enzymes implies that they directly arterialize in BBB damage, which permits inflammatory cell and molecule infiltration, contributing to the worsening of symptoms [19,34]. Similarly, Pleș et al. highlighted that the augmentation of MMP-9 during migraine episodes contributes to both structural disruption of the BBB and heightened neuroinflammatory responses, thus sustaining the migraine process through a feedback loop of vascular and inflammatory activation [46].

One of the possible processes is the growth of the BBB extracellular matrix, rising permeability to meet the required vasodilation, and elevated cerebral circulation during a headache attack. In addition, the same enzyme may cause overactive neural tissue inflammatory responses, which will lead to the development of neuroinflammation because it stimulates the secretion of pro-inflammatory cytokines (TNF-alpha and IL-1beta), which enhance the inflammatory phenomenon and make people more sensitive to pain during headache attacks. Although the activation of MMP-9 has not been definitively proven in migraine patients, the exact mechanisms by which MMP-9 contributes to the development or maintenance of migraine attacks remain unclear. Evidence exists in support of this mechanism based upon animal work, which indicates that it is associated with cortical spreading depression, an electrophysiologic event seen in animal models of migraine with aura [23,35].

Nitric oxide: Among the neurophysiological roles of nitric oxide (NO), it might be a potent vasodilator. Animal models of migraine pathophysiology show that nitric oxide is involved in the condition. Nitroglycerin injection through the formation of NO in rodents induces neuronal activation of various brain areas, among them periaqueductal gray matter, caudal trigeminal nucleus, and the pain genesis. Hypothalamic regions as well as brainstem areas are also stimulated. Furthermore, the injection of nitroglycerin in the trigeminal nucleus facilitates the release of CGRP, substance P, and neuronal nitric oxide synthase (nNOS), which therefore leads to vasodilation. It is assumed that the release of CGRP due to the influence of NO included the participation of transient receptor potential vanilloid (TRPV) receptors; however, not all mechanisms are still clear [36]. Similarly, Karsan et al. demonstrated that NO donors such as nitroglycerin reliably provoke migraine-like attacks in both animal and human models, thereby reinforcing the significance of the NO-CGRP axis in migraine pathophysiology and therapeutic targeting [38].

Molecular Mechanisms of Glucocorticoids in Migraine Symptom Reduction

The glucocorticoid mechanisms are associated with steroidal anti-inflammatory effects or direct involvement in migraine pathophysiology, such as expression of various MMP-9 inhibitors, reduced blood-brain barrier permeability, production of nitric oxide, and reduction of the level of CGRP.

General Anti-inflammatory Mechanisms

Glucocorticoids are strong anti-inflammatory substances that control the immunity and inflammatory reactions through the regulation of various gene transformations. Firstly, they combine with intracellular receptors to create complexes that further move to the cell nuclei to trigger the modifications of gene expressions, causing some inhibition or creation of proteins. Anti-inflammatory actions include preventing certain production of mediators [34-37].

Phospholipase A2 (PLA2) inhibition occurs when early effect modulators of glucocorticoids enhance the expression of lipocortin, also known as annexin 1. The protein is a direct inhibitor of PLA2, and it obstructs the release of arachidonic acid at the cell membranes. In case arachidonic acid is made unreliable, the system of prostaglandins is instantly disconnected, restricting the creation of such mediators.

Cyclooxygenase-2 (COX-2) expression inhibition: Glucocorticoids have a direct influence on the expression of the COX-2 enzyme, which is significantly upregulated during an inflammatory event. Glucocorticoids inhibit the underlying transcription of COX-2 by interfering with pro-inflammatory transcription factors, including nuclear factor kappa B (NF-κB) and activator protein 1 (AP-1). The inhibition of COX-2 leads to a lower mode of transformation of arachidonic acid to the production of prostaglandins and, in turn, leads to a notable reduction of the production of inflammatory molecules.

Increased MAPK phosphatase 1 production (mitogen-activated protein kinase): This increase inhibits phospholipase A2 and MAPK kinase pathways, causing decreased inflammatory gene expression.

Inhibition of other prostaglandin synthesis-stimulating molecules: The level of inflammatory cytokines is also reduced (IL-1B, IL-6, IL-10, TNF-α). Such a complex inflammatory modulation guarantees the control of the production of prostaglandin at several stages and does not allow an inflammatory condition to become worse and cause other symptoms connected to it, like pain and edema.

Migraine is a disease that has neuroinflammation elements, like an increase in the number of mediators in the case of a headache attack that are a part of its pathophysiology [37]. Kursun et al. emphasized that inflammasome activation, particularly through nucleotide-binding domain, leucine-rich-containing family, pyrin domain-containing-3 protein (NLRP3) complexes, contributes to neuroinflammatory signaling in migraine by promoting the release of cytokines that sensitize trigeminal pathways and sustain pain transmission [37].

Specific Mechanisms in Migraine

MMP-9 Expression Inhibition and Decreased BBB Permeability

**Figure 2:**
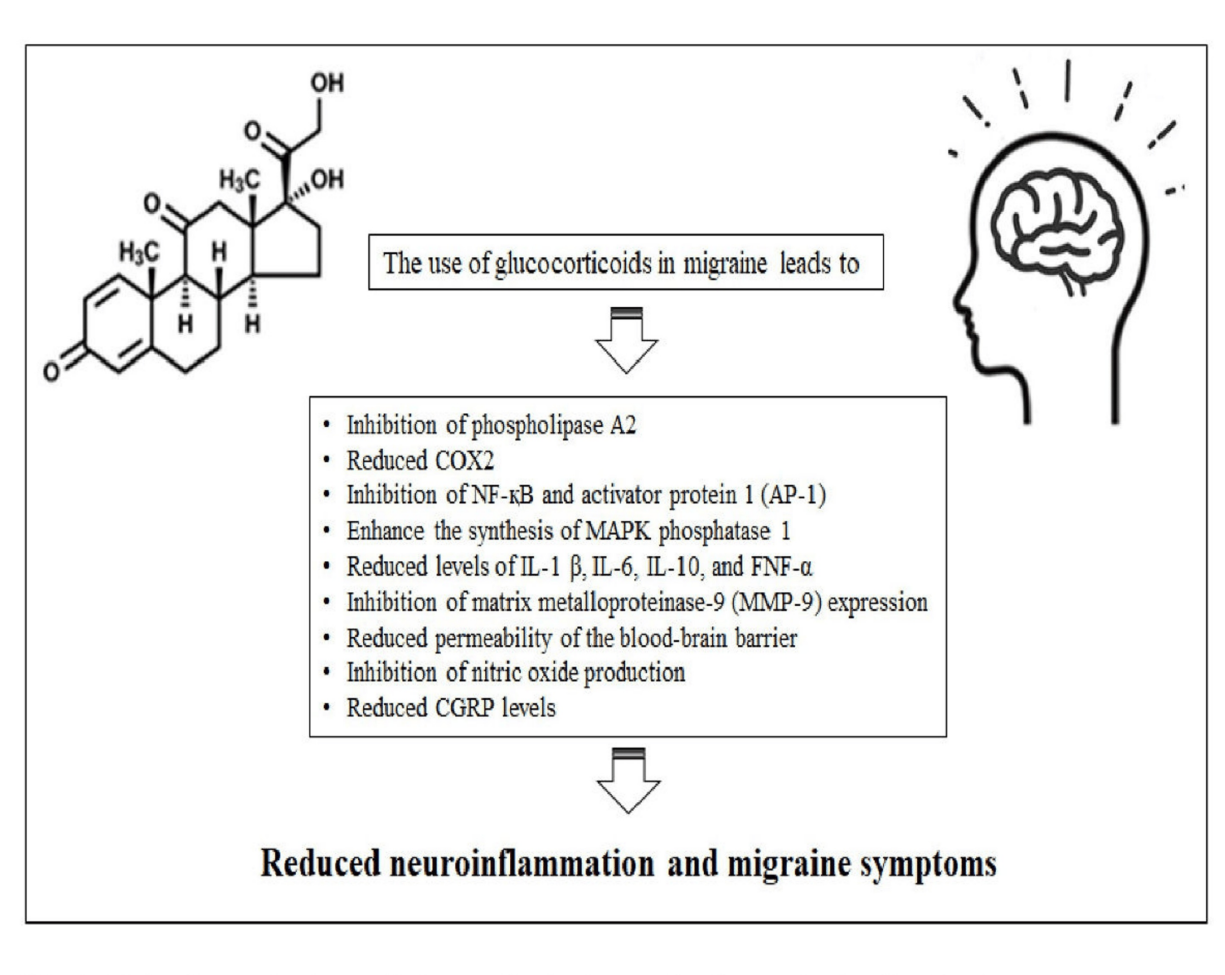
Molecular mechanisms of glucocorticoids that can lead to a reduction in migraine symptoms CGRP: Calcitonin gene-related peptide

The permeability of MMP-9 can be impaired since it has the capacity to break down the basement membranes of capillary endothelial cells, which constitute the BBB. These enzyme levels increase during acute migraine symptoms, both with and without aura. The relationship between MMP-9 levels and migraine development remains unclear; however, research indicates that these enzymes tend to promote neuroinflammatory processes due to increased infiltration of inflammatory cells into the central nervous system and the occurrence of cortical spreading depression. The glucocorticoids are capable of reducing the expression of MMP and increasing the expression of tissue inhibitor of metalloproteinases-1 (TIMP-1), which is an inhibitor of MMP-9. The mechanism can be attributed to improvements in migraine symptoms after the administration of glucocorticoids [19,23,24]. The molecular mechanisms of glucocorticoids are given in Figure 2.

Nitric Oxide Synthesis Inhibition

An aspect of one major glucocorticoid anti-inflammatory effect is the inhibition of NF-κB. This typically causes the transcription of genes that encode mediators, such as cytokines (IL-1, TNF-α) and enzymes like inducible nitric oxide synthase (iNOS), which play roles in the production of nitric oxide (NO). Additionally, the glucocorticoids also regulated other non-pro-inflammatory trans-factors, including AP-1. These processes lead to the reduction in NO synthesis, which is a key player in the pathophysiology of migraine effects as a vasodilator, activator of neuropathways, and useful promoter of CGRP release [24,27,31,32].

CGRP Level Reduction

High CGRP can serve as a biomarker and be directly linked with the pathophysiology of pain. CGRP is vital when activating the trigeminal system and leads to processes such as vasodilation, neurogenic inflammation, and the transmission of pain signals. Additionally, the release of CGRP from nerve endings activates glial cells, induces elevated levels of cytokines (TNF-alpha and IL-1 beta), and sustains inflammatory cycles that can lead to central sensitization. The increase in this cytokine production in other inflammatory cells, too, has been reported [32,33].

The IL-1beta is an inflammatory factor that shows direct action on the release of CGRP in cultures of trigeminal ganglion cells in in vitro experiments. Glucocorticoids alter IL-1 by lowering its production and slowing its effects on the inflammatory cells. Glucocorticoids achieve this by inhibiting the down-regulation of inflammation-related gene expression, including IL-1 and IL-1beta, thereby regulating their levels towards normalcy for migraine patients. CGRP is reduced, and suppression of neural pathways involved in migraine pathophysiology further reduces cytokine formation. Clinical research indicates that CGRP is diminished during an attack of headaches with the use of glucocorticoids [27,32].

Study Limitations

This review has several limitations. First, the heterogeneity in study populations, treatment regimens, dosages, and outcome measures restricts the ability to generalize findings. Then, many included studies had methodological weaknesses such as small sample sizes, lack of blinding, or incomplete follow-up, which may introduce bias. Furthermore, mechanistic insights were often derived from animal or in vitro studies, which may not fully replicate human migraine pathophysiology. Finally, publication bias cannot be excluded, as studies with negative or inconclusive results may be underrepresented. These limitations point to the need for well-designed, large-scale clinical trials and translational studies to better define the therapeutic role of glucocorticoids in migraine.

## Conclusions

Migraine is a complex neuroinflammatory disorder involving multiple interacting pathways, including CGRP signaling, PACAP regulation, oxidative stress, nitric oxide production, and MMP-9 activation. These pathways collectively contribute to vasodilation, neuropeptide release, and central sensitization, supporting recurrent and potentially progressive inflammation. Glucocorticoids may modulate several of these mechanisms, but their clinical effects are generally modest. They are not first-line abortive treatments but may provide adjunctive benefit in severe, acute, or refractory cases, especially when individualized by patient characteristics, timing, and dose. Future research should explore predictive biomarkers and combination therapies targeting multiple molecular pathways. Tailored anti-inflammatory strategies could improve outcomes in chronic or treatment-resistant migraine.

## Appendices

**Table 3:** Database-specific search strategies

Database	Search Strategy (2004–2024; English)
PubMed (via MEDLINE)	("Migraine Disorders"[MeSH Terms] OR migraine* OR "migraine medications") AND (glucocorticoid* OR corticosteroid* OR dexamethasone OR prednisolone OR prednisone) AND ("2004/01/01"[Date - Publication] : "2024/03/31"[Date - Publication])
SciELO	(migraine OR "migraine medications") AND (glucocorticoids OR corticosteroids OR dexamethasone OR prednisolone OR prednisone) Filters: 2004–2024; English language
LiLacs	(mh:"migraine disorders" OR migraine) AND (mh:"glucocorticoids" OR corticosteroid* OR dexamethasone OR prednisolone OR prednisone) AND (mh: "drug therapy" OR "acute treatment") Publication years: 2004–2024
Bireme (VHL Regional Portal)	(tw:(migraine)) AND (tw:(glucocorticoids OR corticosteroids OR dexamethasone OR prednisolone OR prednisone)) AND (tw:("acute treatment" OR pharmacological therapy)) Limits: 2004–2024; English
ScienceDirect	TITLE-ABSTR-KEY(migraine OR "migraine medications") AND TITLE-ABSTR-KEY(glucocorticoids OR corticosteroids OR dexamethasone OR prednisolone OR prednisone) AND PUBYEAR > 2003 AND PUBYEAR < 2025 Filters: Research articles, Review articles, English

**Table 4:** Methodological quality of the articles included

Authors & Year	Selection	Exposure Ascertained	Outcome Ascertained	Alternative Causes Ruled Out	Challenge/Rechallenge	Dose–Response	Follow-up Adequate	Detailed Reporting
Colman et al., 2008	Yes	Yes	Yes	Yes	No	No	Yes	Yes
Giuliano et al., 2012	NA	NA	NA	NA	NA	NA	NA	Yes
Silvestro et al., 2023	NA	NA	NA	NA	NA	NA	NA	Yes
Krymchantowski et al., 2020	Yes	Yes	Yes	Yes	No	No	No	Yes
Huang et al., 2013	Yes	Yes	Yes	Yes	No	No	No	Yes
Silva-Néto et al., 2014	Yes	Yes	Yes	Yes	No	No	No	Yes
Villar-Martinez et al., 2022	NA (Review)	NA	NA	NA	NA	NA	NA	Yes
Russo et al., 2023	NA	NA	NA	NA	NA	NA	NA	Yes
Ferrari, 2022	NA	NA	NA	NA	NA	NA	NA	Yes
Edvinsson, 2021	NA	NA	NA	NA	NA	NA	NA	Yes
Kamm, 2022	NA	NA	NA	NA	NA	NA	NA	Yes
Chan et al., 2019	NA	NA	NA	NA	NA	NA	NA	Yes
Jiménez-Jiménez et al., 2024	NA	NA	NA	NA	NA	NA	NA	Yes
Gupta, 2009	NA	NA	NA	NA	NA	NA	NA	Yes
Karademir et al., 2022	Yes	Yes	Yes	Yes	No	No	No	Yes
Gursoy-Ozdemir, 2004	NA	Yes	Yes	Yes	No	No	No	Yes
Rhen et al., 2005	NA	NA	NA	NA	NA	NA	NA	Yes
Vandewalle et al., 2018	NA	NA	NA	NA	NA	NA	NA	Yes
Williams, 2018	NA	NA	NA	NA	NA	NA	NA	Yes
Kursun, 2021	NA	NA	NA	NA	NA	NA	NA	Yes
Kim, 2008	NA	Yes	Yes	Yes	Yes	No	No	Yes
Karsan, 2023	NA	NA	NA	NA	NA	NA	NA	Yes
Tfelt-Hansen et al., 2009	Yes	Yes	Yes	Yes	No	No	Yes	Yes
Neeb et al., 2016	NA	Yes	Yes	Yes	No	No	No	Yes
Cuesta et al., 2002	NA	Yes	Yes	Yes	No	No	No	Yes
Neeb et al., 2014	Yes	Yes	Yes	Yes	No	No	Yes	Yes

**Table 5:** Methodological quality of the articles included

Cochrane Risk of Bias 2 tool for RCTs								
Study ID	Randomization process	Deviations from interventions	Missing outcome data	Measurement of outcome	Selection of reported results	Overall Risk of Bias		
Tfelt-Hansen et al., 2009 [19]	Low	Low	Low	Low	Low	Low		
ROBINS-I assessment of non-randomized studies								
Study ID	Confounding	Selection of participants	Classification of interventions	Deviations from interventions	Missing data	Measurement of outcomes	Selection of reported result	Overall Risk of Bias
Krymchantowski et al., 2020 [16]	Serious	Moderate	Low	Low	Low	Moderate	Moderate	Serious
Silva-Néto et al., 2014 [17]	Low	Low	Low	Low	Low	Low	Low	Low
Karademir et al., 2022 [18]	Moderate	Moderate	Low	Low	Low	Moderate	Moderate	Moderate
Neeb et al., 2014 [20]	Serious	Moderate	Low	Low	Moderate	Moderate	Moderate	Serious
SYRCLE’s Risk of Bias Tool for animal (in vivo) studies.								
Study ID	Sequence generation	Allocation concealment / Random housing	Blinding (caregivers/researchers/outcome)	Incomplete outcome data	Selective reporting	Reproducibility / Methods detail	Overall Risk of Bias	
Gursoy-Ozdemir, 2004 [22]	Some concerns	High	High	Low	Low	Some concerns	Some concerns	
Kim, 2008 [23]	Low	Some concerns	Some concerns	Low	Low	Low	Low	
Neeb et al., 2016 [24]	NA (in vitro)	NA	High	Low	Some concerns	Some concerns	Some concerns	
Cuesta et al., 2002 [25]	NA (in vitro)	NA	High	Some concerns	Some concerns	Some concerns	Some concerns	
Narrative reviews (based on SANRA — Scale for the Assessment of Narrative Review Articles)								
Study	Clear research aim	Justification of topic importance	Literature search described	Referencing quality	Scientific reasoning	Balanced presentation of evidence	Overall Quality	
Silvestro et al., 2023 [11]	Yes	Yes	Partial (not systematic)	High	High	Balanced	High	
Giuliano et al., 2012 [15]	Yes	Yes	Partial (limited search details)	Moderate	Moderate	Slight emphasis on supportive studies	Moderate	
Villar-Martinez et al., 2022 [26]	Yes	Yes	No (narrative synthesis only)	Moderate	Moderate	Balanced	Moderate	
Russo et al., 2023 [27]	Yes	Yes	Partial	High	High	Balanced	High	
Ferrari, 2022 [28]	Yes	Yes	No	Moderate	Moderate	Slightly selective	Moderate	
Edvinsson, 2021 [29]	Yes	Yes	Partial	High	High	Balanced	High	
Kamm, 2022 [30]	Yes	Yes	No	Moderate	Moderate	Balanced	Moderate	
Chan et al., 2019 [31]	Yes	Yes	Partial	High	High	Balanced	High	
Jiménez-Jiménez et al., 2024 [32]	Yes	Yes	No	Moderate	Moderate	Slight emphasis on oxidative stress	Moderate	
Gupta, 2009 [33]	Yes	Yes	No	Low–Moderate	Low	Selective	Low	
Rhen et al., 2005 [34]	Yes	Yes	No	Moderate	Moderate	Balanced	Moderate	
Vandewalle et al., 2018 [35]	Yes	Yes	Partial	High	High	Balanced	High	
Williams, 2018 [36]	Yes	Yes	Partial	High	High	Balanced	High	
Kursun, 2021 [37]	Yes	Yes	No	Moderate	Moderate	Balanced	Moderate	
Karsan, 2023 [38]	Yes	Yes	Partial	High	High	Balanced	High	

**Table 6:** Methodological quality of the articles included (AMSTAR-2)

AMSTAR-2 (A Measurement Tool to Assess Systematic Reviews)																
Authors & Year	D1: Protocol registered before review	D2: Study design explained	D3: Comprehensive literature search	D4: Duplicate study selection	D5: Duplicate data extraction	D6: List of excluded studies & justification	D7: Characteristics of included studies reported	D8: Risk of bias from individual studies assessed	D9: Sources of funding for included studies reported	D10: Appropriate methods for meta-analysis	D11: Risk of bias considered in interpretation	D12: Adequacy of heterogeneity discussion	D13: Publication bias investigated	D14: Conflict of interest declared	D15: Selection of studies explained	D16: Overall confidence (AMSTAR-2 rating)
Colman et al., 2008 [14]	Yes	Yes	Yes	Yes	Yes	Partial (not all excluded studies listed)	Yes	Yes	No (funding of included trials not reported)	Yes	Yes	Yes	Partial (publication bias not fully addressed)	Yes	Yes	High (minor limitations)
Huang et al., 2013 [21]	No (protocol not explicitly registered)	Yes	Yes	Yes	Yes	Partial	Yes	Yes	Partial	Yes	Yes	Yes	Yes	Yes	Yes	Moderate (some critical domains unclear)
